# Sample entropy reveals an age-related reduction in the complexity of dynamic brain

**DOI:** 10.1038/s41598-017-08565-y

**Published:** 2017-08-11

**Authors:** Yanbing Jia, Huaguang Gu, Qiang Luo

**Affiliations:** 1 School of Aerospace Engineering and Applied Mechanics, Tongji University, Shanghai, 200092 P. R. China; 2 School of Life Sciences, Fudan University, Shanghai, 200433 P. R. China; 3 Institute of Science and Technology of Brain-Inspired Intelligence, Fudan University, Shanghai, 200433 P. R. China

## Abstract

Dynamic reconfiguration of the human brain is characterized by the nature of complexity. The purpose of this study was to measure such complexity and also analyze its association with age. We modeled the dynamic reconfiguration process by dynamic functional connectivity, which was established by resting-state functional magnetic resonance imaging (fMRI) data, and we measured complexity within the dynamic functional connectivity by sample entropy (SampEn). A brainwide map of SampEn in healthy subjects shows larger values in the caudate, the olfactory gyrus, the amygdala, and the hippocampus, and lower values in primary sensorimotor and visual areas. Association analysis in healthy subjects indicated that SampEn of the amygdala-cortical connectivity decreases with advancing age. Such age-related loss of SampEn, however, disappears in patients with schizophrenia. These findings suggest that SampEn of the dynamic functional connectivity is a promising indicator of normal aging.

## Introduction

Complexity is one of the defining natures of the brain. Brain signals measured by neuroimaging techniques reflect the collective activities of populations of neurons with different rhythms. Because the rhythm of neurons can be periodic, chaotic, or random^1–3^, brain signals would show different levels of complexity, which has been demonstrated by entropy analysis of blood oxygenation level–dependent (BOLD) signals^4–9^. Recently, neuroimaging studies have begun to reveal the dynamic reconfiguration of brain functional architecture using dynamic functional connectivity (i.e., time-varying correlations in BOLD signals between distinct brain regions across different time windows) and to demonstrate its relevance to cognitive functions and diseases^10–12^. As the complexity within the BOLD signal varies across different brain regions^4, 5^, we expect to see a distribution of the complexity within the dynamic functional connectivity of different brain regions. However, the complexity within the dynamic functional connectivity has yet to be quantitatively characterized.

Apart from complexity, several methods have been proposed to measure different aspects of dynamic functional connectivity^12–15^. For example, patterns of the functional connectivity were clustered into distinct classes, and the functional reconfiguration among these classes was modeled as a Markov chain^13^. To characterize the temporal variability of a brain region, resting-state BOLD signals were segmented into nonoverlapping windows and the functional connectivity patterns of this region were compared among these windows^15^. These methods, however, cannot estimate the complexity within the dynamic functional connectivity (see the supplementary information). Entropy is a well-defined statistical concept used to measure the complexity within dynamic processes, with larger entropy corresponding to greater complexity^16^. Given the limited number of sampling points in an functional magnetic resonance imaging (fMRI) experiment, the classical estimations of the entropy are inaccurate^5, 6^. Sample entropy (SampEn)^17^ can overcome this drawback and has been used commonly in entropy analysis of fMRI data^5, 6, 18^.

As a universal and secular phenomenon, the aging process has been suggested to be a progressive loss of complexity within the dynamics of physiologic outputs^19^. This age-related loss is believed to stem from the gradual deterioration of the underlying structural components of physiological systems and from alterations within the coupling between these systems^20^. Previous studies have shown that the complexity within the temporal dynamics of brain signals measured by electroencephalography (EEG), magnetoencephalography (MEG), and BOLD fMRI appears to decrease^18, 20–23^. Because the dynamic functional connectivity also is a typical physiologic output of the brain, we expected that the complexity within the dynamic functional connectivity of some brain regions also would decrease with advancing age.

In schizophrenia, nonlinearity has been assumed to be underlying the irregularity in psychotic symptoms^24^. A large body of studies on electroencephalography (EEG) and magnetoencephalography (MEG) reported increased or reduced complexity in patients with schizophrenia compared with healthy controls. Such divergence might be modulated by symptomatology, age effects, and so on (for a review, see Fernandez^25^). More important, not only schizophrenia^26^ but also some other mental diseases, such as Alzheimer’s disease^27^, major depression^28^, and attention deficit-hyperactivity disorder^29^, disrupt the normal evolution of complexity as a function of age. If complexity of dynamic functional connectivity is a promising indicator of normal aging, we would expect a disrupted association between complexity and age in patients with schizophrenia.

In the present study, we used SampEn to (1) quantitatively characterize the complexity within the dynamic functional connectivity, (2) identify dynamic functional connectivity showing an age-related reduction in complexity during normal aging, and (3) test whether these age-related reductions are disrupted by schizophrenia.

## Materials and Methods

### Participants

The study included 62 healthy subjects (25 males and 37 females) and 69 patients with chronic schizophrenia (35 males and 34 females). Exclusion criteria included the presence of DSM-IV Axis I diagnoses of other disorders such as bipolar disorder, history of any substance dependence, or history of clinically significant head trauma. Participants’ demographic characteristics are shown in Table 1. This dataset was selected from our previous resting-state fMRI study, which included multicenter datasets^30^, and was suitable to investigate the aging process because the age of healthy subjects and the age of patients with schizophrenia were both uniformly distributed across a relatively wide range. The age of healthy subjects was from 19 to 51 years (mean ± SD, 29.87 ± 8.62 years) and the degree of education was from 6 to 21 years (mean ± SD, 15.29 ± 2.39 years). One of the healthy subjects was left-handed, and the others were right-handed. The healthy subjects were assessed in accordance with DSM-IV criteria as being free of schizophrenia. The age of patients with schizophrenia was from 17 to 55 years (mean ± SD, 31.95 ± 9.60 years) and the degree of education was from 9 to 18 years (mean ± SD, 14.19 ± 2.16 years). Two of the patients were left-handed, and the others were right-handed. Illness durations of the patients ranged from a few months to 30 years (mean ± SD, 7.17 ± 6.61 years). Symptom severity was measured using the Positive and Negative Syndrome Scale (PANSS) assessment, which was given to all patients either one week before or one week after the MRI scan. Five patients, however, were not able to complete their PANSS assessment because of their poor health condition. The healthy and patient groups were well matched by gender (*χ* ^2^ = 1.4234, *P* = 0.2328) and age (t-test, *P* = 0.2836), although the healthy subjects had a slightly longer education degree (t-test, *P* = 0.0064). All of the research procedures and ethical guidelines were in accordance with the Institutional Review Board (IRB) of the National Taiwan University Hospital. We obtained written informed consent from all individual participants, and IRB of the National Taiwan University Hospital approved this study.

**Table 1:** Demographic information for the participants. PANSS, Positive and Negative Syndrome Scale.

	Healthy subjects (*n* = 62)	Patients with schizophrenia (*n* = 69)	*P* value
Age (year)	29.87 ± 8.62	31.95 ± 9.60	0.2836
Education (year)	15.29 ± 2.39	14.19 ± 2.16	0.0064
Sex (M/F)	25/37	35/34	0.2328
Illness duration (year)	—	7.17 ± 6.61	—
PANSS-positive scale	—	11.92 ± 4.71	—
PANSS-negative scale	—	13.61 ± 6.33	—
PANSS-general scale	—	27.28 ± 9.64	—

### Image acquisition

Scanning was performed on a 3 T Siemens Trio Tim MRI scanner. FMRI images were acquired using a gradient-echo echo planar imaging (GE-EPI) sequence. The following parameters were used: repetition time (TR), 2000 ms; echo time (TE), 24 ms; field of view (FOV), 256 × 256 mm^2^; matrix, 64 × 64; slice thickness, 3 mm; flip angle (FA), 90°. For each subject, 34 transaxial slices with no gap were acquired to cover the whole brain volume. Each scan contained 180 volumes. During the scan, participants were instructed to relax, remain calm, keep their eyes closed, and refrain from thinking about anything in particular.

### Data preprocessing

The fMRI data were preprocessed using Statistical Parametric Mapping (SPM8; http://www.fil.ion.ucl.ac.uk/spm) and Data Preprocessing Assistant for Resting-state fMRI (DPARSF)^31^. The first 10 volumes were discarded to allow for scanner stabilization and subject adaption to the scanning environment. The remaining functional scans were corrected for delay in slice acquisition and interscan head movement. Subsequently, the functional scans were spatially normalized to the stereotactic space (Montreal Neurological Institute) and resampled to 3 mm isotropic voxels. All normalized images were then smoothed with an 8 mm Gaussian kernel. The BOLD signal of each voxel was then linearly detrended and passed through a band-pass filter (0.01–0.08 Hz). Nuisance covariates were regressed out using multiple linear regression, including six head-motion parameters, white matter signals, cerebrospinal signals, and global mean signals. We chose global signal removal as it has been shown to reduce physiological noise and the variance because of movement-related effects^32, 33^. To reduce the effect of head motion, we further conducted careful volume censoring (“scrubbing”) movement correction^34^. The mean framewise displacement (FD) was calculated with an FD threshold of 0.5 mm for exclusion. Except for the frame corresponding to the displaced time point, we also removed one preceding and two succeeding time points to reduce the spillover influence of head motion. All participants with >10% displaced frames or who exhibited more than 3° of maximal rotation or 3 mm of maximal translation were completely excluded from the following analysis.

### Dynamic functional connectivity analysis

**Figure 1:**
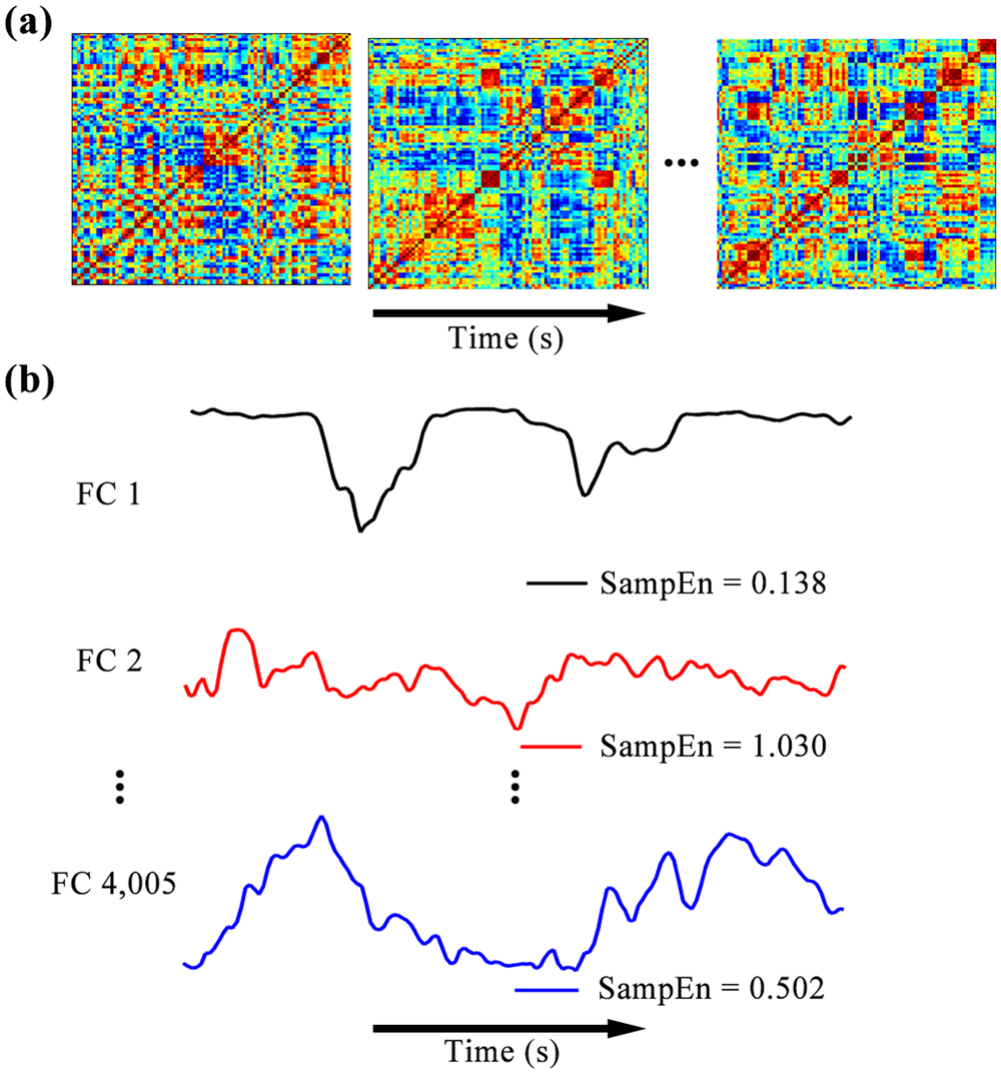
SampEn analysis of dynamic functional connectivity. Dynamic functional connectivity matrices at different time windows for one subject (**a**). We extracted the functional connectivity time series between each pair of ROIs from these dynamic functional connectivity matrices, and evaluated SampEn of each functional connectivity time series (**b**). FC, functional connectivity.

We used the automated anatomical labeling atlas^35^ to divide the human brain into 90 relatively large regions of interests (ROIs). The time series was extracted in each ROI by averaging the BOLD signals of all voxels within that ROI. This approach reduces noise contained in these BOLD signals and commonly has been used in resting-state fMRI studies^15, 30, 36^. The names and abbreviations of the ROIs are listed in Supplementary Table S1. For each subject, we performed dynamic functional connectivity analysis using the sliding window approach^13^. The time window with a length of 20 TRs (40 s) slid in steps of 1 TR^12^, resulting in 150 windows. Given a time window, we evaluated the functional connectivity between each pair of ROIs using Pearson correlation. Thus, we obtained dynamic functional connectivity matrices for each subject (Fig. 1a). The functional connectivity time series between each pair of ROIs then was extracted from these matrices (Fig. 1b). We selected the window length on the basis of the observations that window sizes in the range of 30–60 s produce robust results in cognitive states^37^, and variations of functional connectivity are not sensitive to the specific window size in the range of 20–40 s^12, 38^.

Pearson correlation only captures linear associations between time series and often results in negative correlations. Indeed, we found that the mean proportion of negative correlations across all dynamic functional connectivity matrices of all participants was 0.4998 ± 0.0134 (mean ± SD). Because we were mainly interested in the dynamic property of the functional connectivity, we did not deal with these negative correlations and directly calculated SampEn of each functional connectivity time series (Fig. 1b).

### SampEn analysis of dynamic functional connectivity

We denote a time series of length *N* by *x* = [*x*_1_, *x*_2_, …, *x*_*N*_]. SampEn of the time series can be calculated as follows^5, 6^.

First, construct an embedding vector with *m* consecutive data points extracted from *x*: *v*_*i*_ = [*x*_*i*_, *x*_*i*+1_, …, *x*_*i+m*−1_], where *m* is the embedding dimension.

Second, define for each *i* (1 ≤ *i* ≤ *N* − *m*)

1 $${C}_{i}^{m}\,=\,\frac{1}{N-m-1}\sum _{j=1,\,j\ne i}^{N-m}{\rm{\Theta }}(r-{\Vert {v}_{i}-{v}_{j}\Vert }_{1}).$$

Here, *r* specifies a tolerance value and *r* = *εσ*_*x*_, where *ε* is a scaling parameter and *σ*_*x*_ is the standard deviation of *x*. ${\rm{\Theta }}(\cdot )$ is the Heaviside function:

2 $${\rm{\Theta }}(x)=\,\{\begin{array}{c}0,\,x < 0\\ 1,\,x\ge 0\end{array},$$

and ${\Vert \cdot \Vert }_{1}$ represents Chebyshev distance, that is,

3 $${\Vert {v}_{i}-{v}_{j}\Vert }_{1}=\,{\rm{\max }}(|{x}_{i}-{x}_{j}|,\,|{x}_{i+1}-{x}_{j+1}|,\,\ldots \,,\,|{x}_{i+m-1}-{x}_{j+m-1}|).$$

That is, ${C}_{i}^{m}$ represents the proportion of *v*_*j*_ (*j* ≠ *i*) whose distances to *v*_*i*_ are less than *r*. Similarly, for each *i* (1 ≤ *i* ≤ *N* − *m*), we also define

4 $${C}_{i}^{m+1}=\,\frac{1}{N-m-1}\sum _{j=1,\,j\ne i}^{N-m}{\rm{\Theta }}(r-{\Vert {v}_{i}-{v}_{j}\Vert }_{1})\,,$$

where ${C}_{i}^{m+1}$ represents the proportion corresponding to the dimension of *m* + 1; ${C}_{i}^{m}$ and ${C}_{i}^{m+1}$ have the same form, but embedding vectors in the two cases are defined in different phase spaces.

Third, by averaging across all embedding vectors, we get

5 $${U}^{m}=\,\frac{1}{N-m}\sum _{i=1}^{N-m}{C}_{i}^{m},$$

and

6 $${U}^{m+1}\,=\,\frac{1}{N-m}\sum _{i=1}^{N-m}{C}_{i}^{m+1}.$$

Fourth, SampEn of *x* is calculated as

7 $${\rm{SampEn}}=-\mathrm{ln}({U}^{m+1}/{U}^{m}).$$

Following these four steps, we obtained SampEn of each functional connectivity time series for each subject (Fig. 1b). SampEn assigned a nonnegative number to each functional connectivity time series, with larger values corresponding to more complexity or irregularity in the time series^16^. It was suggested that, for *m* = 1 or 2 and *ε* in the range of 0.1–0.25, SampEn shows good statistical properties^18, 39^. Throughout this study, *m* was fixed at 2 as it was shown that *m* = 2 enables more detailed reconstruction of the joint probabilistic dynamics of the time series^40^. In the main analysis, *ε* was fixed at 0.2.

For each subject, after SampEn of each functional connectivity time series was obtained, we further calculated SampEn of ROIs. We obtained SampEn of a given ROI by averaging SampEn of 89 functional connectivity time series corresponding to it. We also calculated SampEn of resting state networks (RSNs). Previous studies showed that the whole brain can be divided into six RSNs^41^, which can be classified as follows: a default mode network (RSN 1), an attention network (RSN 2), a visual recognition network (RSN 3), an auditory network (RSN 4), a sensorimotor network (RSN 5), and a subcortical network (RSN 6). Supplementary Table S2 lists the ROIs in each RSN. We obtained SampEn of a given RSN by averaging SampEn of all ROIs contained in it.

### Association analysis between SampEn and age in healthy subjects

We used partial correlation coefficients to investigate associations between SampEn and age, conditioning on gender, education, and head motion. We performed the partial correlation analyses sequentially at three different levels of the brain. First, we obtained partial correlation coefficients of all RSNs. Second, for each RSN significantly identified in the first step, we calculated partial correlation coefficients of all ROIs contained in this RSN. Third, for each ROI that showed a significant effect in the second step, we obtained partial correlation coefficients of all functional connections corresponding to this ROI. We applied false-positive discovery rate (FDR) controls to correct for multiple comparisons in each of the three steps. The dimensions of the correction in the first step and the third step were the number of RSNs (=6) and the number of functional connections corresponding to an ROI (=89), respectively. Because the number of ROIs in different RSNs is different, the dimension of the correction in the second step was not a fixed constant.

### Association analysis between SampEn and age in patients with schizophrenia

To test whether the schizophrenic disorder alters the associations between SampEn and age, in patients with schizophrenia, we also performed association analyses of the functional connections that were identified as significant in the association analysis between SampEn and age in healthy subjects. We also used partial correlation coefficients to investigate these associations between SampEn and age, conditioning on gender, education, and head motion. For each functional connection, using the webpage http://vassarstats.net/rdiff.html, we calculated a value of *Z* that could be applied to assess the significance of the difference between the partial correlation coefficient of the healthy group and that of the patient group. To correct for multiple comparisons, we applied the FDR control. The dimension of the correction was the number of functional connections identified as significant in the association analysis between SampEn and age in healthy subjects.

To study whether the recognized deviations from the normal aging are related to schizophrenia, we studied associations between SampEn of the functional connections, which show significant group differences in partial correlation coefficients, and clinical variables including symptom severity scores (i.e., PANSS) and illness duration. Partial correlation coefficients were used to investigate these associations, conditioning on age, gender, education, and head motion.

### Data Availability

The datasets generated during the current study are available from the corresponding author on reasonable request.

## Results

### SampEn varies throughout the brain

**Figure 2:**
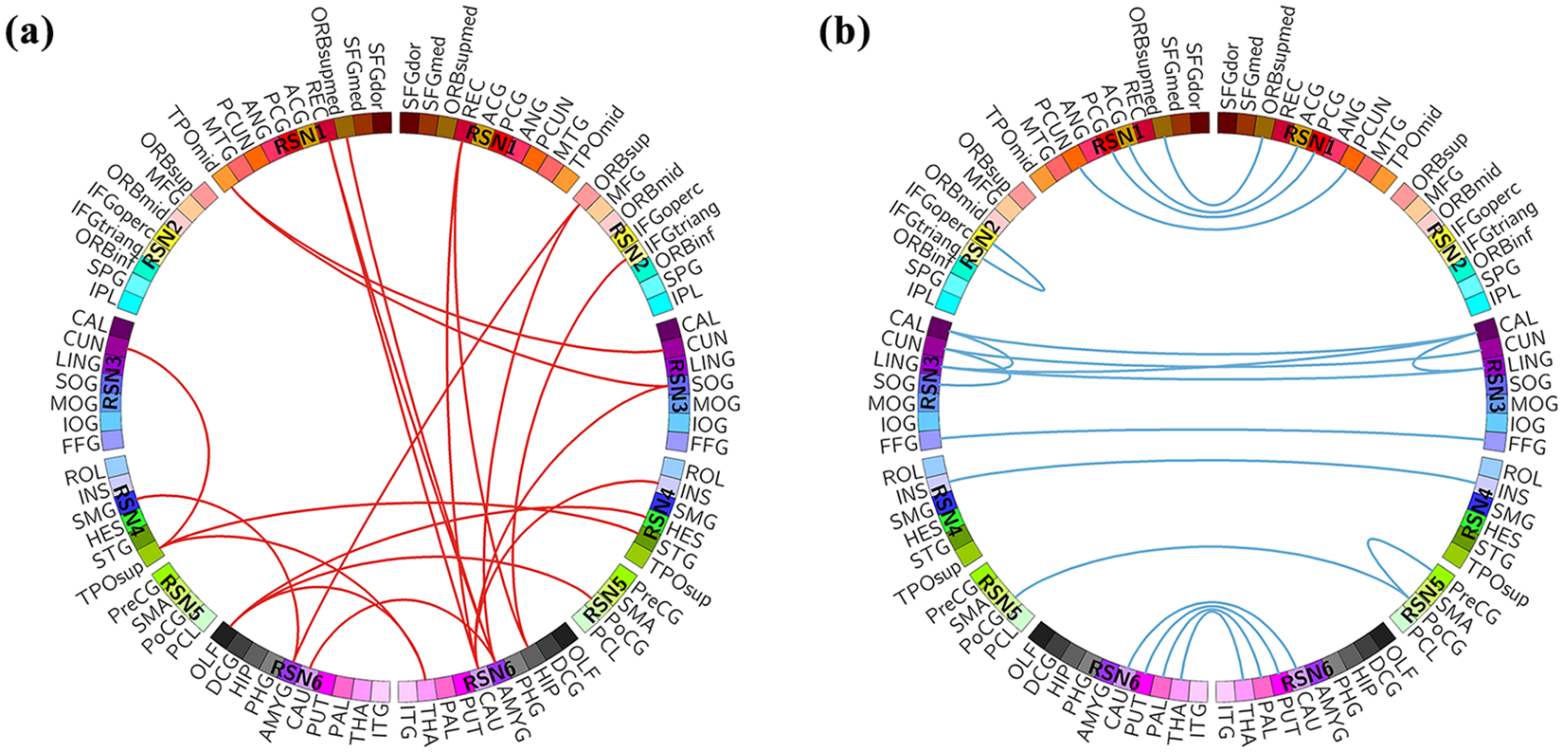
Connectogram of functional connections with the largest and the lowest 0.5% SampEn. Functional connections with the largest SampEn (**a**). Functional connections with the lowest SampEn (**b**). The left part and the right part of the connectogram represent the left hemisphere and the right hemisphere of the brain, respectively. Supplementary Table S1 lists the full name of each ROI.

We quantified SampEn of each functional connection by the mean SampEn averaged across all healthy subjects. Figure 2 shows the functional connections among 90 ROIs with the largest and the lowest 0.5% SampEn. Supplementary Dataset 1 also lists the mean SampEn of each functional connection in descending order. We found that functional connections implicated in higher cognitive functions (e.g., memory, emotion, and reward) show larger SampEn, including the fronto-limbic system (e.g., the fronto-hippocampal and the fronto-amygdala connectivity) and the fronto-striatal system (e.g., the rectus-caudate connectivity). In contrast, functional connections in sensorimotor cortices (e.g., the lingual gyrus, the calcarine gyrus, the precentral gyrus, and the postcentral gyrus) show lower SampEn. We also found that the homotopic interhemispheric connections show lower SampEn such as functional connections of the postcentral gyrus, the thalamus, the calcarine gyrus, and the lingual gyrus.

**Figure 3:**
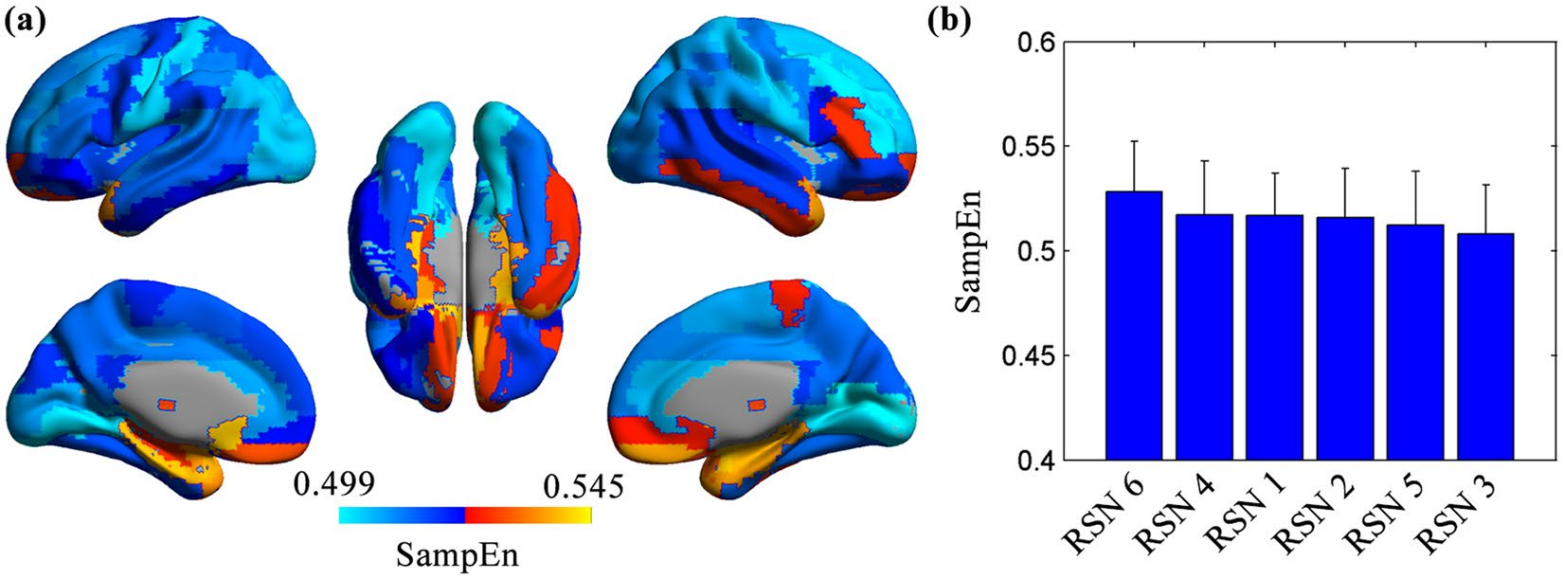
The mean SampEn of each ROI and each RSN across all healthy subjects. The mean SampEn of each ROI across all healthy subjects (**a**). The color bar indicates the values of SampEn. The mean SampEn of each RSN across all healthy subjects is depicted in descending order (**b**). The error bars represent SD. RSN1, the default mode network; RSN 2, the attention network; RSN 3, the visual recognition network; RSN 4, the auditory network; RSN 5, the sensorimotor network; RSN6, the subcortical network.

The mean SampEn of each ROI across all healthy subjects is shown in Fig. 3a and also is listed in descending order in Supplementary Dataset 2. We found that the limbic system and its adjacent areas show higher SampEn (e.g., the caudate, the olfactory gyrus, the amygdala, the parahippocampus, and the hippocampus), whereas sensorimotor cortices (e.g., the lingual gyrus, the calcarine gyrus, and the postcentral gyrus) demonstrate lower SampEn. The mean SampEn of each RSN across all healthy subjects is shown in Fig. 3b. We found that the subcortical network and the auditory network show higher SampEn, whereas the sensorimotor network and the visual recognition network demonstrate lower SampEn.

### SampEn decreases during the normal aging process

**Figure 4:**
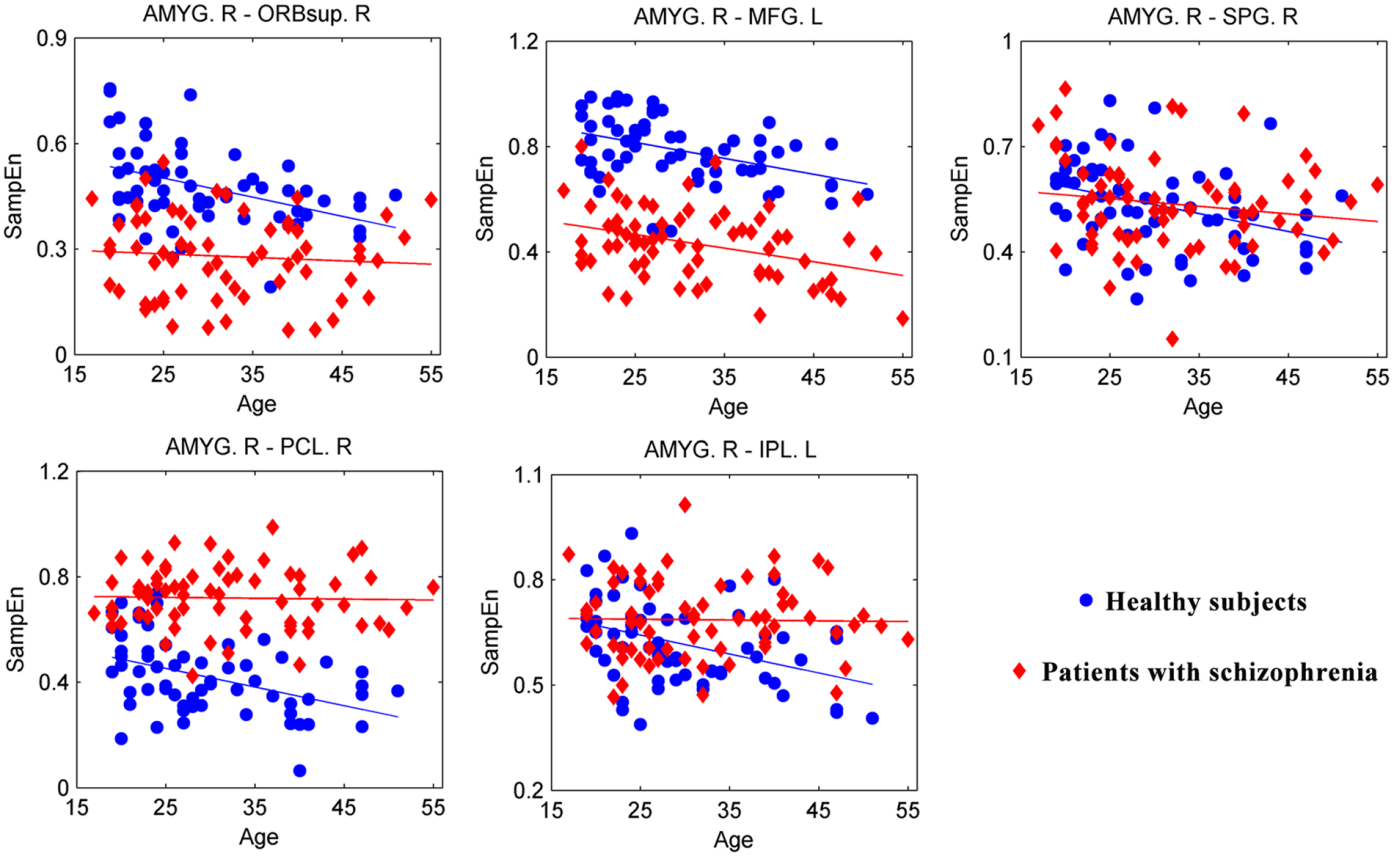
Associations between age and SampEn of functional connections in healthy subjects and patients with schizophrenia. AMYG, amygdala; ORBsup, superior orbital frontal gyrus; MFG, middle frontal gyrus; SPG, superior parietal gyrus; PCL, paracentral lobule; IPL, inferior parietal gyrus; L, left; R, right.

First, we detected a negative association between age and SampEn of the subcortical network in healthy subjects (*P* = 0.0022, *R* = −0.3744, FDR corrected; Table 2 and Supplementary Fig. S2). Second, for ROIs in the subcortical network, we found that SampEn of the bilateral amygdala, the right pallidum, the bilateral thalamus, and the left caudate negatively associates with age (*P* < 0.05, FDR corrected; Table 2 and Supplementary Fig. S2). Finally, for functional connections corresponding to these six ROIs in the subcortical network, we identified negative associations between age and SampEn of the functional connectivity between the right amygdala and the right superior orbital frontal gyrus, the left middle frontal gyrus, the right superior parietal gyrus, the right paracentral lobule, and the left inferior parietal gyrus (*P* < 0.05, FDR corrected; Table 3 and Fig. 4).

**Table 2:** Associations between age and SampEn of RSNs and ROIs in healthy subjects. *R* is the partial correlation coefficient. RSN 6, the subcortical network; AMYG, amygdala; PAL, pallidum; THA, thalamus; CAU, caudate; L, left; R, right.

RSNs and ROIs	*R*	*P* value
*RSNs*		
RSN6	−0.3744	0.0022
*ROIs*		
AMYG. R	−0.4234	0.0006
PAL. R	−0.3753	0.0022
AMYG. L	−0.3694	0.0025
THA. L	−0.3704	0.0025
THA. R	−0.3592	0.0033
CAU. L	−0.3005	0.0122

**Table 3:** Associations between age and SampEn of functional connections in healthy subjects and patients with schizophrenia. For the difference between *R*_SZ_ and *R*_HC_, *P* values that could survive FDR correction are marked in bold. HC, healthy subjects; SZ, patients with schizophrenia; *R*_HC_, partial correlation coefficient of the healthy group; *R*_SZ_, partial correlation coefficient of the patient group; AMYG, amygdala; ORBsup, superior orbital frontal gyrus; MFG, middle frontal gyrus; SPG, superior parietal gyrus; PCL, paracentral lobule; IPL, inferior parietal gyrus; L, left; R, right.

Functional connections	HC		SZ		*R*_SZ_ > *R*_HC_	
	*R*_HC_	*P* value	*R*_SZ_	*P* value	*Z* value	*P* value
AMYG. R - ORBsup. R	−0.4459	0.0003	0.0159	0.5488	**2.73**	**0.0052**
AMYG. R - MFG. L	−0.4241	0.0006	−0.2489	0.0255	1.09	0.1379
AMYG. R - SPG. R	−0.4204	0.0006	−0.0751	0.2809	**2.06**	**0.0197**
AMYG. R - PCL. R	−0.4044	0.0010	−0.0378	0.3852	**2.16**	**0.0154**
AMYG. R - IPL. L	−0.3847	0.0017	−0.0304	0.4073	**2.07**	**0.0192**

### Schizophrenia uncouples SampEn and age

For these five functional connections that show significant effects in healthy subjects, SampEn of four functional connections is disassociated with age (*P* > 0.05; Table 3 and Fig. 4), including the functional connectivity between the right amygdala and the right superior orbital frontal gyrus, the right superior parietal gyrus, the right paracentral lobule, and the left inferior parietal gyrus. For each of these four functional connections, the partial correlation coefficient of the patient group is significantly larger than that of the healthy group (*P* < 0.05, FDR corrected; Table 3). Further association analysis between SampEn and clinical variables showed that SampEn of the functional connectivity between the right amygdala and the right superior orbital frontal gyrus is associated with the illness duration, whereas SampEn of the functional connectivity between the right amygdala and the left inferior parietal gyrus is associated with the general scale and the illness duration (Table 4).

**Table 4:** Associations between SampEn of functional connections and clinical variables. *R* is the partial correlation coefficient. *P* values less than 0.05 are marked in bold. AMYG, amygdala; ORBsup, superior orbital frontal gyrus; SPG, superior parietal gyrus; PCL, paracentral lobule; IPL, inferior parietal gyrus; L, left; R, right.

Functional connections	Positive scale		Negative scale		General scale		Illness duration	
	*R*	*P* value	*R*	*P* value	*R*	*P* value	*R*	*P* value
AMYG. R - ORBsup. R	−0.1329	0.1537	−0.0931	0.2378	0.1168	0.1851	**0.2596**	**0.0208**
AMYG. R - SPG. R	0.0757	0.2811	0.0936	0.2365	0.1620	0.1062	−0.1766	0.0849
AMYG. R - PCL. R	0.0259	0.4216	−0.0112	0.4660	−0.0628	0.3155	0.0058	0.4820
AMYG. R - IPL. L	0.1711	0.0937	0.0800	0.2701	**0.3106**	**0.0075**	−**0.2759**	**0.0150**

## Discussion

In summary, we applied SampEn to characterize the complexity within the dynamic functional connectivity. This is different from previous studies on the complexity within BOLD signals^4–9^. Such characterization is important because it enables us to investigate the biological implications of changes in brain functional architecture. As a demonstration, we established a SampEn map of the dynamic brain, and identified an age-related reduction in SampEn, suggesting that not only SampEn of the activation in some brain regions but also SampEn of the dynamic functional connectivity evolves with age. Moreover, we found that the age-related reduction in SampEn could be altered by schizophrenia, calling for further research of schizophrenia on the dynamics of the amygdala-cortical functional connections, which show significant alterations in our study.

The brain is a complex dynamic system, and how to characterize the dynamics in the brain is nontrivial. In the current study, we proposed the use of a well-defined measure named SampEn to characterize the complexity within the dynamic functional connectivity estimated by applying the sliding-window correlation technique. The dynamical functional connectivity, however, might be induced by nonneuronal signals, including cardiac and respiratory signals. This may cause potential problems in many measures characterizing dynamic properties of the dynamic functional connectivity, especially when these confounding processes are periodic, because a periodic signal is considered to be variable regardless of its amplitude^42^. Unlike these previous studies, we analyzed SampEn of the dynamic functional connectivity. According to the physical interpretation of SampEn, larger SampEn corresponds to greater irregularity in the dynamic functional connectivity. Because periodic signals are characterized by regularity, SampEn of periodic signals is relatively low, and thereby, we could control the confounding effects of periodic signals in our analysis.

Remarkably, SampEn map of the dynamic brain captures the functionalities of different brain regions reported in the literature. We found that most of the brain regions with high SampEn have been implicated in key aspects of learning. For instance, the caudate is implicated in reinforcement-based associative learning^43^ and classification learning^44^, the olfactory cortex in olfactory learning^45^, the amygdala in emotional learning^46^, the hippocampus in many aspects of learning and memory^47^, and the temporal pole in visual learning^48^. In contrast, we observed that sensorimotor and visual areas show low SampEn. These results add SampEn of the dynamic functional connectivity as a new aspect to the literature, reporting that flexibility of the brain is an important factor predicting learning^49^ and that primary sensorimotor and visual areas show low flexibility during motor learning task^50^.

Aging is a fundamental process in the human brain, and deviations from the normal aging process would result in mental disorders. With advancing age, the complexity within the temporal dynamics of brain signals measured by EEG, MEG, and BOLD fMRI appears to decrease^18, 20–23^. Consistently, we found that SampEn of the dynamic brain also decreases with advancing age. These decreases may relate to the decline in capacity of learning and memory with normal aging that is caused by alterations in neuronal structure and losses of synapses^51^. Moreover, we identified that the reduction in SampEn of the dynamic brain is driven mainly by the amygdala-cortical functional connectivity. The amygdala-cortical connectivity has been reported to be age-related in both structural^52^ and functional^53, 54^ neuroimaging studies, but the current study revealed the negative association between SampEn of the amygdala-cortical functional connectivity and age. This result is complementary to a previous study that showed that increased resting-state functional connectivity between the amygdala and frontal regions is related to superior emotional regulation in aging^55^.

Notably, the proposed approach is clinically relevant. A most recent study reported that the subcortical-cortical resting-state functional connectivity decreases with advancing age in the general population, and this age-related decrease is insignificant in patients with autism^56^. These results suggest that the normal aging process is altered by mental disorders. In the current study, we found a similar deviation from the normal aging process as the association between SampEn and age significantly decreases in patients with schizophrenia. Interestingly, the most significant deviation was identified in SampEn of functional connectivity between the right amygdala and the right superior orbital frontal cortex. This is consistent with a previous study that reported the association between the decreased amygdala-frontal functional connectivity and emotional abnormalities in patients with schizophrenia^57^. Moreover, we found that SampEn of the functional connectivity between the right amygdala and the right superior orbital frontal gyrus is lower in healthy elderly subjects compared with healthy young subjects, and is also lower in patients with schizophrenia compared with healthy subjects (Supplementary Fig. S3). This finding provides new evidence for the accelerated aging in patients with schizophrenia, which already has been demonstrated by previous studies that have reported an accelerated decline in functional brain network efficiency^58^ and an accelerated reduction of gray matter volume^59^.

A prominent advantage of our approach is that SampEn of the dynamic functional connectivity does not necessarily depend on the scaling parameter *ε*. As shown in Supplementary Fig. S4, SampEn of the dynamic functional connectivity obtained at a different value of *ε* (e.g., 0.12, 0.16, and 0.24) was highly correlated with that obtained at 0.2 (*R* > 0.97). The interpretation of findings in the present study, however, needs to consider some limitations. Because noise also shows high SampEn according to the physical interpretation of SampEn, the observational noise in BOLD signals may contribute to SampEn of the dynamic functional connectivity. In the current study, we have associated SampEn with age, which suggests that the contribution of the observational noise to SampEn is not significant. Additionally, because medical treatment data were not available in the current sample, future analyses on patients with schizophrenia with records of medical treatment would help us to assess the confounding effect of the medical treatment on our findings.

## Electronic supplementary material

Supplementary Information

Dataset 1

Dataset 2
