# Supplementary material for: Sample entropy reveals an age-related reduction in the complexity of dynamic brain
Source: Sci Rep. 2017 Aug 11;7:7990. doi: 10.1038/s41598-017-08565-y (PMC5554148; doi:10.1038/s41598-017-08565-y)
Supplement: Supplementary file 1 — Supplementary Information [file 41598_2017_8565_MOESM1_ESM.pdf]

Supplementary Information

Sample entropy reveals an age-related reduction in the complexity of dynamic brain

Yanbing Jia[1], Huaguang Gu[1,*] and Qiang Luo[2,3,*]

[1] School of Aerospace Engineering and Applied Mechanics, Tongji University, Shanghai 200092, P.R. China

[2] School of Life Sciences, Fudan University, Shanghai 200433, P. R. China

[3] Institute of Science and Technology of Brain-Inspired Intelligence, Fudan University, Shanghai 200433, P. R. China

[*]Correspondence should be addressed to the following addresses:

Qiang Luo, School of Life Sciences, Fudan University, Shanghai 200433, P. R. China, E-mail: qluo@fudan.edu.cn, Tel: +86-21-65648454, Fax: +86-21-65648454.

Or

Huaguang Gu, School of Aerospace Engineering and Applied Mechanics, Tongji University, Shanghai 200092, P. R. China, E-mail: guhuaguang@tongji.edu.cn, Tel: +86-21-65983627, Fax: +86-21-65983627.

Supplementary Text

Comparison of SampEn and variability by numerical simulation

Many measures have been proposed to characterize different aspects of the dynamic brain. However, how to interpret such characteristics is currently under debate, as nonneuronal signals (e.g., cardiac and respiratory signals) can induce such variations[1,2]. One of the most recent examples is the variability proposed by Zhang *et al.*[3] measuring the temporal variation in the profile of the functional connectivity between a region to all the other regions in the brain. To demonstrate the difference between variability and SampEn, we used the following numerical simulations.

First, we simulated three types of signals, including periodic, chaotic, and mixed signals. We generated mixed signals based on periodic and chaotic signals. We simulated periodic signals using the following algebraic equation:

$$x_1(t) = \sin\left(\frac{\pi}{25}t + \varphi\right), \tag{S1}$$

where φ is a random variable in the interval [0, 2π]. We generated chaotic signals using the following Duffing equation:

$$\frac{d^2 x_2(t)}{dt^2} + 0.3\frac{dx_2(t)}{dt} - x_2(t) + x_2^3(t) = 0.32\cos(1.2t). \tag{S2}$$

Different random initial conditions were used to generate different chaotic solutions. We simulated mixed signals using the following algebraic equation:

$$x_3(t) = x_1(t) + \mu x_2(t), \tag{S3}$$

where μ is a parameter in the range [0, 1].

Second, to model dynamic functional connectivity, $x_1(t)$, $x_2(t)$, and $x_3(t)$ were resampled with the sampling period TR = 2 s and then normalized to the interval [−1, 1].

Third, assuming that dynamic functional connections corresponding to brain regions A, B, and C were modeled by the resampled signals of $x_1(t)$, $x_2(t)$, and $x_3(t)$, respectively.

Fourth, SampEn and variability of the regions A, B, and C were obtained. The final results were generated by averaging the 20 independent runs to warrant

appropriate statistical accuracy with respect to the stochastic simulations.

Variability of the regions A and B was 1.1328 ± 0.0006 (mean ± SD) and 0.9410 ± 0.0243, respectively. SampEn of the regions A and B was 0.2538 ± 0.0006 and 0.9199 ± 0.0238, respectively. Thus, both the regions A and B showed high variability (≈ 1), whereas SampEn of the region B was about three times of SampEn of the region A. The dependence of SampEn and variability of the region C on μ is shown in Supplementary Fig. S1. As μ is increased, SampEn increases dramatically, whereas variability maintains around 1. These simulation results indicate that SampEn and variability are two remarkably different indices.

Additionally, results in the present study are also different from results in Zhang *et al.*[3]. We recognized that the limbic system and its adjacent areas show the highest SampEn, whereas Zhang *et al.* identified that the middle/inferior temporal gyrus shows the highest variability.

References

1 Calhoun, V., Yaesoubi, M., Rashid, B. & Miller, R. *IEEE Global Conference on Signal and Information Processing,* 831-834 (2013).

2 Hutchison, R. M. *et al.* Dynamic functional connectivity: promise, issues, and interpretations. *Neuroimage* **80**, 360-378 (2013).

3 Zhang, J. *et al.* Neural, electrophysiological and anatomical basis of brain-network variability and its characteristic changes in mental disorders. *Brain* **139**, 2307-2321 (2016).

Supplementary Figures

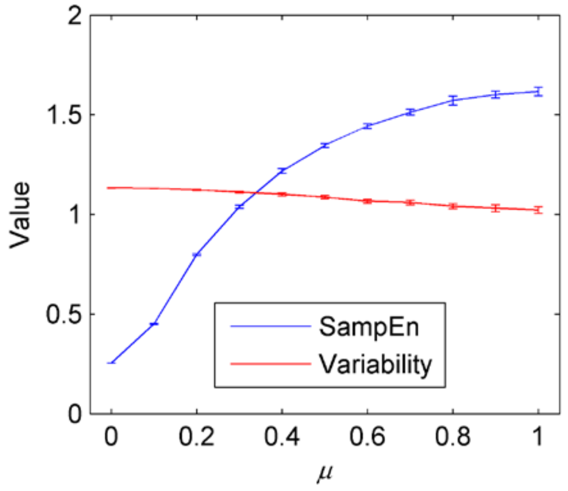

Figure S1. The dependence of SampEn and variability of the region C on μ. The error bars represent SD.

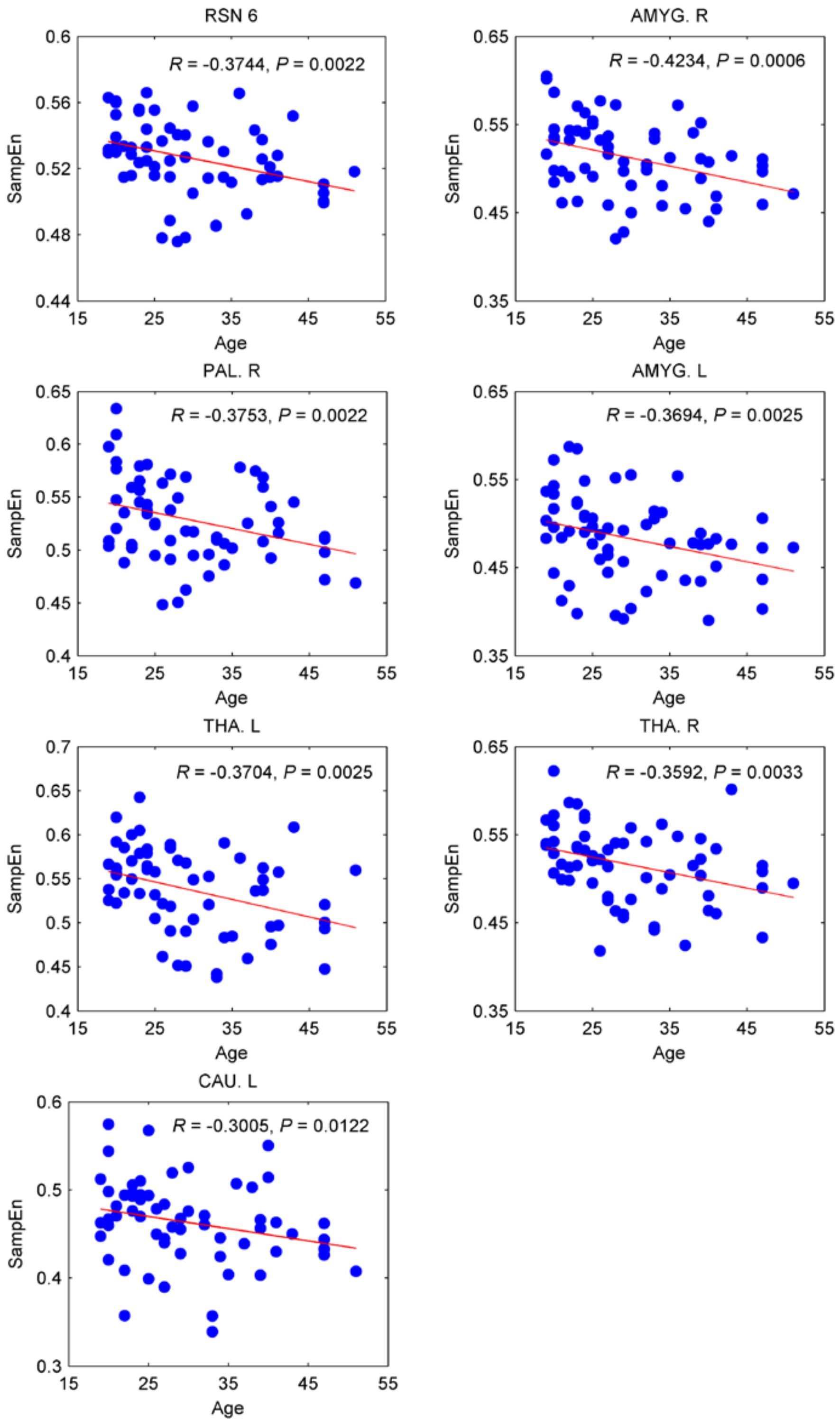

Figure S2. Associations between age and SampEn of RSNs and ROIs in healthy subjects. The linear fit is shown where *R* is the partial correlation coefficient and *P* is the significance level. RSN 6, the subcortical network; AMYG, amygdala; PAL, pallidum; THA, thalamus; CAU, caudate; L, left; R, right.

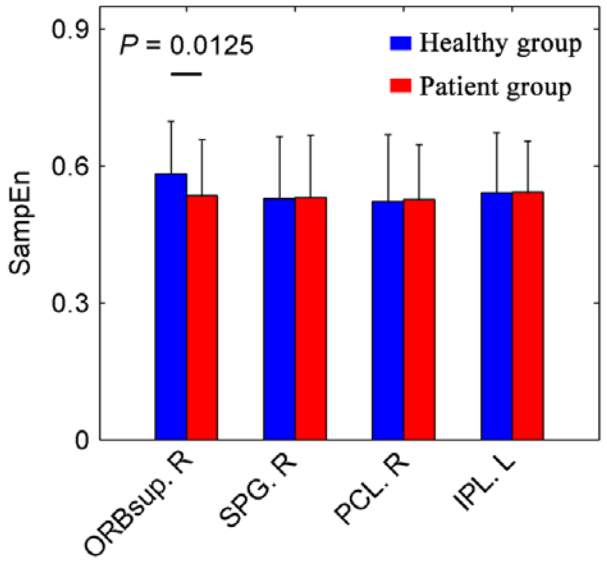

Figure S3. Bar chart of the mean SampEn of the functional connections listed in Table 4. SampEn of the functional connectivity between the right amygdala and the right superior orbital frontal gyrus shows significant group difference (P = 0.0125, FDR corrected). The dimension of the correction is four. The error bars represent SD. ORBsup, superior orbital frontal gyrus; SPG, superior parietal gyrus; PCL, paracentral lobule; IPL, inferior parietal gyrus; L, left; R, right.

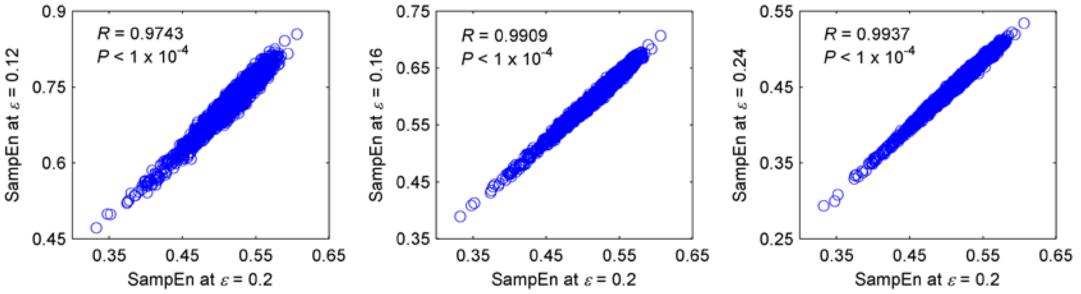

Figure S4. Correlation between SampEn at $\varepsilon = 0.2$ and that at a different value of ε. Each circle represents a functional connection, and the SampEn is averaged across all healthy subjects. *R* is the Pearson correlation coefficient and *P* is the significance level.

Supplementary Tables

No.	Regions	Abbr.	No.	Regions	Abbr.
1, 2	Precental gyrus	PreCG	47, 48	Lingual gyrus	LING
3, 4	Superior frontal gyrus, dorsolateral	SFGdor	49, 50	Superior occipital gyrus	SOG
5, 6	Superior frontal gyrus, orbital part	ORBsup	51, 52	Middle occipital gyrus	MOG
7, 8	Middle frontal gyrus	MFG	53, 54	Inferior occipital gyrus	IOG
9, 10	Middle frontal gyrus, orbital part	ORBmid	55, 56	Fusiform gyrus	FFG
11, 12	Inferior frontal gyrus, opercular part	IFGoperc	57, 58	Postcentral gyrus	PoCG
13, 14	Inferior frontal gyrus, triangular part	IFGtriang	59, 60	Superior parietal gyrus	SPG
15, 16	Inferior frontal gyrus, orbital part	ORBinf	61, 62	Inferior parietal	IPL
17, 18	Rolandic operculum	ROL	63, 64	Supramarginal gyrus	SMG
19, 20	Supplementary motor area	SMA	65, 66	Angular gyrus	ANG
21, 22	Olfactory cortex	OLF	67, 68	Precuneus	PCUN
23, 24	Superior frontal gyrus, medial	SFGmed	69, 70	Paracentral lobule	PCL
25, 26	Superior frontal gyrus, medial orbital	ORBsupmed	71, 72	Caudate nucleus	CAU
27, 28	Gyrus rectus	REC	73, 74	Lenticular nucleus, putamen	PUT
29, 30	Insula	INS	75, 76	Lenticular nucleus, pallidum	PAL
31, 32	Anterior cingulate and paracingulate gyri	ACG	77, 78	Thalamus	THA
33, 34	Median cingulate and paracingulate gyri	DCG	79, 80	Heschl gyrus	HES
35, 36	Posterior cingulate gyrus	PCG	81, 82	Superior temporal gyrus	STG
37, 38	Hippocampus	HIP	83, 84	Temporal pole: superior temporal gyrus	TPOsup
39, 40	Parahippocampal gyrus	PHG	85, 86	Middle temporal gyrus	MTG
41, 42	Amygdala	AMYG	87, 88	Temporal pole: middle temporal gyrus	TPOmid
43, 44	Calcarine fissure and surrounding cortex	CAL	89, 90	Inferior temporal gyrus	ITG
45, 46	Cuneus	CUN			

Table S1. The names and abbreviations of ROIs.

RSN	ROI	RSN	ROI
RSN 1	SFGdor	RSN 4	ROL
	SFGmed		INS
	ORBsupmed		SMG
	REC		HES
	ACG		STG
	PCG		TPOsup
	ANG	RSN 5	PreCG
	PCUN		SMA
	MTG		PoCG
	TPOmid		PCL
RSN 2	ORBsup	RSN 6	OLF
	MFG		DCG
	ORBmid		HIP
	IFGoperc		PHG
	IFGtriang		AMYG
	ORBinf		CAU
	SPG		PUT
	IPL		PAL
RSN 3	CAL		THA
	CUN		ITG
	LING		
	SOG		
	MOG		
	IOG		
	FFG		

Table S2. ROIs in each RSN. Supplementary Table S1 lists the full name of each ROI. RSN 1, the default mode network; RSN 2, the attention network; RSN 3, the visual recognition network; RSN 4, the auditory network; RSN 5, the sensorimotor network; RSN 6, the subcortical network.
